# Point‐Of‐Care Ultrasound in Emergency Departments in Australia/New Zealand: An Emergency Physician's Perspective

**DOI:** 10.1002/jmrs.871

**Published:** 2025-03-05

**Authors:** Robyn Brady

**Affiliations:** ^1^ Staff Specialist Emergency Physician, Clinical Lead Ultrasound, Lismore Base Hospital Lismore New South Wales Australia; ^2^ Queensland Children's Hospital South Brisbane Queensland Australia

## Abstract

This brief overview of the current state of clinician performed focused ultrasound (Emergency PoCUS) by emergency practitioners in Australia/New Zealand (ANZ) has touched on its history, scope of practice both mandated and context‐dependent, complex embedding in clinical diagnostic reasoning and range of governance issues. It is the author's hope that an ongoing understanding and interplay between the three professional groups most closely involved in the use of ultrasound to improve patient care and health‐care flow can continue to work closely together for the ultimate benefit of patients in multiple contexts in ANZ and beyond.

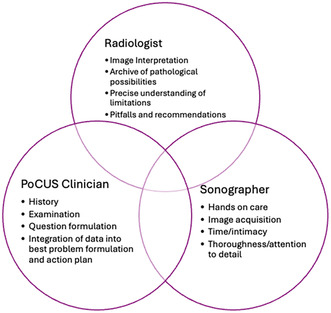

## 1 Introduction

Ever since the ‘Focused Abdominal Sonography in Trauma’ (FAST) exam transformed emergency medicine practice with the concept of immediate, clinician‐performed point‐of‐care ultrasound (PoCUS) as a clinical tool to seek rapid, high specificity evidence of significant blunt trauma [1], emergency medicine has been exploring and extending the role of clinician‐performed portable ultrasound within its operations. This ‘disruptive innovation’ has taken a variety of names and forms in different contexts and countries, with attendant benefits, risks and implications for groups from patients, clinicians, administrators, educators to medical imaging specialists including sonographers. This editorial presents a review of the terminology, evolution, contexts, unique features and current state of governance for PoCUS in Emergency Medicine from the viewpoint of an Australian emergency specialist in the college‐defined, ‘Clinical Lead of Ultrasound’ (CLUS) role.

## 2 What's in a Name?

Point of care ultrasound (PoCUS) was defined in a *New England Journal of Medicine* (NEJM) article in 2011 as advanced diagnostic ultrasonography that is performed and interpreted by the attending physician as a bedside test [2]. Emergency medicine ultrasound (EMUS) describes ‘the application of bedside PoCUS by the attending emergency physician to assist in the diagnosis and management of any time‐sensitive health emergencies’ [3]. Within the Australian College of Emergency Medicine (ACEM) and the Australasian Society of Ultrasound in Medicine (ASUM) position statements, the term ‘focused ultrasound’ is preferred [4, 5]. The American College of Radiologists have defined and differentiated point‐of‐care ultrasound use by characteristics such as how/why images are sought, whether technical assistance is provided in image acquisition, and how or whether images are identified, labelled, archived and reported [6]. A detailed assessment of various definitions, components and alliances is given in a 2025 review of PoCUS governance in the USA [7]. While in Australia, Emergency PoCUS use does not attract a Medicare rebate, stakeholders monitoring these evolving characteristics report to the Diagnostic Imaging Stakeholder Committee which advises the Department of Health Aged Care on matters relating to the Medical Benefits Schedule [8].

PoCUS is the primary search term available through PubMed to gather and disseminate research in this rapidly evolving area, with over 3500 articles triggered by the search criteria ‘point‐of‐care ultrasound AND emergency’ in the last 5 years alone. The clinical context for PoCUS is now extensive, ranging from family medicine and pre‐hospital care to ophthalmology, rheumatology, gastroenterology, respiratory medicine and other clinical specialties, as well as the more established users in obstetrics and cardiology, so the term Emergency PoCUS will be used here to describe focused ultrasound performed by clinicians in the emergency department.

## 3 Emergency PoCUS in the Clinical Setting

Aside from its use in improving the accuracy of needle procedures, which is long established [9], there are three broad ‘diagnostic’ usages of Emergency PoCUS, requiring different levels of skill and governance, from the use of the handheld probe as an extension of the physical examination much like the stethoscope (stony dullness/pleural effusion yes/no, breath sounds yes/no, central pulse present yes/no) [10, 11, 12], through standard emergency applications discussed below, to occasional use in more diagnostically and sonographically challenging and risk‐laden constructs such as the acute abdomen [3].

Similarly but not identically, there are different levels of ultrasound specific certification in use, ranging from no additional certification beyond undergraduate or specialty training, to the two ASUM levels of credentialing: the well‐established CCPU standard (Certificate of Clinician Performed Ultrasound‐ eg., [13]) and the less common Diploma of Diagnostic Ultrasound‐ Emergency (DDU) [14], a clinician‐sonologist who can perform, report and archive focused comprehensive imaging while still within the context defined by the diagnosis and management of time‐sensitive health emergencies. The new ACEM defined internal credentialing guidelines [15] are modelled on the CCPU and define current best practice standards for institutional credentialing for the middle usage above, that is, Emergency PoCUS applications to aid diagnostic streaming in time critical situations. Many specialties, such as the College for Intensive Care Medicine, now mandate equivalent level ultrasound training for all their trainees [16].

## 4 Historical and Situational Considerations

**FIGURE 1:**
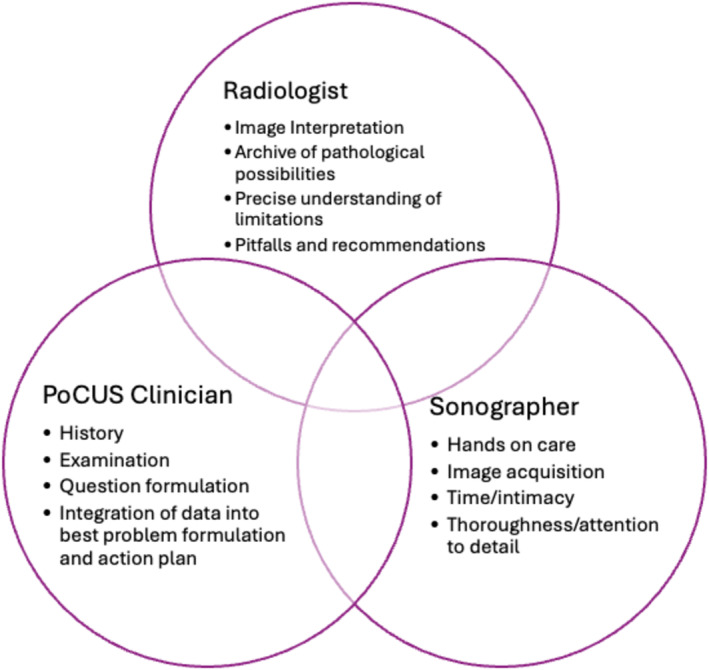
Relationship of clinician to sonographer and Radiologist with primary roles and contextual and evolving overlaps.

The components of an Emergency PoCUS examination include (1) evaluation of the clinical situation and formulation of a diagnostic reasoning schema to which specific data outcomes from imaging or pathology tests can contribute (2) ultrasound image acquisition, involving physics, eye hand coordination, extensive experience and hands‐on physical and relational management of the body of an often anxious and suffering person [17] (3) image interpretation, including reference to past imaging, known age and gender dependent norms, possible further imaging and a detailed understanding of the likelihood and implications of any conclusions and (4) integration of multiple pieces of imperfect information into the clinical management to create a new diagnostic schemata or ‘best problem formulation’ and plan for that person at that point in time and physical context. In Australia, components two and three have been traditionally performed by sonographers and radiologists, respectively, as shown in Figure 1.

The evolution of ultrasound in clinical practice has occurred somewhat differently in other countries and specialties. As pioneers in ultrasound, obstetricians and ophthalmologists have always performed ultrasound themselves [18].

In some European and Asian countries, the professional sonographer does not exist or has been a recent development, and radiologist preference for ‘quicker’, higher resolution modalities with greater diagnostic yield, plus early access to ultrasound machines by emergency physicians and generalists led to adoption of clinician performed bedside ultrasound in the 1990s, and a long history of integration into medical education and specialty training [3].

Areas with limited radiologist access, particularly prior to/without digital transfer capacities privileged the development of sonographer reporting as occurs in parts of New Zealand, UK and South Africa and is described in Portugal [19, 20]. Sonographer reporting can have accuracy similar to that of radiology [20, 21] and in the right hands and contexts can be invaluable [19, 22]. In contexts with limited access to sonographers or echocardiographers, the ability for Emergency PoCUS trained clinicians to perform more detailed sonographic evaluations can also fast track appropriate treatment, and while the ASUM DDU (Emergency) and CCPU programs are designed to provide the necessary structured experience and skills for this, there are many examples of uncredentialed Emergency PoCUS clinicians fast‐tracking patients with time critical pathology to centres with appropriate treatment options [23]. As Figure 1 shows, there is context dependent overlap in the roles of these professions—radiologists do obtain sonographic images themselves to clarify pathological differentials, and both radiologists and sonographers can recognise clinical priorities that extend the scope of a clinician's questions and/or over‐ride the pursuit of complete image sets or pathological certainty.

## 5 Current Australasian Emergency PoCUS in Practice

The first ACEM Ultrasound policy was established in 1999 [24] but its widespread adoption only reached a tipping point in Australia with the publication of COR 742 outlining the requirements for Emergency PoCUS education and governance and producing high quality open‐access training modules covering five initial fundamental areas, all modelled on existing CCPU applications: Focused Echo in Life Support (FELS), Extended Focused Abdominal Sonography for Trauma (EFAST), Lung, Abdominal Aortic Aneurysm (AAA) and Ultrasound Guided Procedures [4].

Procedural use aside, these applications all seek to answer a specific relevant clinical question, which is usually posed in a binomial manner, in a focused rather than comprehensive way and integrated as a branch point into a conceptual diagnostic algorithm intended to produce a best problem formulation and prioritise one or another immediate management decisions rather than to necessarily arrive at a final definitive diagnosis. Examples of this abound in critically ill patients and help immediate prioritisation without negating the need for further detailed/comprehensive imaging. The ability to immediately rule out a pneumothorax, or recognise the likelihood of right mainstem bronchus intubation in new hypoxia is game‐changing, and the presence or absence of a generalised B line pattern (suggesting increased interstitial fluid), a heart with decreased left ventricular contractility, and a distended IVC with decreased respiratory variation changes the management and outcomes of patients with septic shock while it is evolving [25, 26].

These assessments often occur in challenging situations with multiple priorities and patient management activities, and without the ability for active patient cooperation or movement and sometimes with limited access to specific desirable sonographic windows.

Increasingly required by state health organisations and/or local institutions as part of contractual scope of practice, credentialing is generally supervised by the CLUS and other Emergency PoCUS credentialed emergency faculty. Self‐guided learning during the logbook phase is enhanced by technical advances in newer Emergency PoCUS machines, which allow simultaneous educational graphic displays and feedback on achieved images [27]. Sonographers have played a key role in modelling governance, education and training [28, 29] including the evolution of a new sonographic sub‐specialty role, the Sonography Educator in the Emergency Department (SEED) [30, 31].

## 6 Medicolegal Risk and Governance

Legal action regarding Emergency PoCUS use remains surprisingly low, a review of civil action in the United States to end 2021 still included only lawsuits for non‐use of Emergency PoCUS in life threatening illness [32, 33]. Insights however have come from collated hospital and medical defence association cases in Canada, which include adverse outcomes from non‐use of Emergency PoCUS (37%) but also from a variety of misuse errors including diagnostic process errors such as anchoring bias, incorrect approach, inadequate skill, documentation or reporting [34]. Within the Australasian Emergency PoCUS community, anecdotal errors have occurred due to misunderstanding the limited significance of negative Emergency PoCUS findings, inadequate differentials for positive findings and failure to communicate significant positive findings through robust mechanisms [23, 35].

The adoption of Emergency PoCUS education into medical school training in Australasia has followed the trajectory seen in North America and Europe, with ultrasound often incorporated into learning of anatomy and physiology as well as clinical process flows such as critical care ‘shock’ teaching. However, the use of Emergency PoCUS in emergency departments requires much more than kinaesthetic skills: a clinically based awareness of priorities and questions, excellent diagnostic reasoning, a constant awareness of potential time‐critical clinical processes as well as departmental flow issues, and a deep understanding of a Boolean approach to clinical questions as multiple new, flawed data points are added. The importance of diagnostic processing errors and perhaps a unique susceptibility to ‘image gratification’ or ‘new tool complacence’ must be addressed by sound modelling of the place of Emergency PoCUS in diagnostic reasoning and embedded into Emergency PoCUS teaching and examination processes. This modelling best occurs at the bedside or in case reviews with explicit illustrations of diagnostic modelling including differential diagnosis, pre‐test probabilities and the importance of time critical interventions. These concepts are illustrated in phases 1 and 4 of the components discussed above, and have been incorporated into the new assessment templates used in ACEM Emergency PoCUS credentialing [36].

Efforts to improve quality assurance are now emphasised both by training bodies such as ACEM and ASUM, and in international forums such as the World Federation for Ultrasound in Medicine and Biology (WFUMB) and International Federation of Emergency Medicine (IFEM). Key documents in this regard include the joint Emergency PoCUS Stewardship position paper by the European Society of Emergency Medicine (EuSEM) and the European Society of Ultrasound in Medicine and Biology (EFSUMB) and the 2025 Medical Clinics of North America paper on PoCUS infrastructure [37, 38]. Image archiving and audit are critical aspects of quality assurance recommended by all governing bodies, and at institutions with the highest published Emergency PoCUS accuracies, these elements are well‐established [7, 39, 40]. However they are only beginning to be addressed in Australia at institutional and state levels, due to the personnel and infrastructure investments required for educational and quality assurance provision and credentialing oversight, archiving middleware, storage and EMR linkages. These elements are essential so that the images obtained, and conclusions reached from the imaging and integrated into patient management plans can be communicated to other teams, reviewed following subsequent developments and subjected to the continuous quality assurance that is required for optimal quality in patient care decision‐making.

## 7 Conclusion

This brief overview of the current state of clinician performed focused ultrasound (Emergency PoCUS) by emergency practitioners in Australia/New Zealand has touched on its history, scope of practice both mandated and context‐dependent, complex embedding in clinical diagnostic reasoning and range of governance issues. It is the author's hope that an ongoing understanding and interplay between the three professional groups most closely involved in the use of ultrasound to improve patient care and health‐care flow can continue to work closely together for the ultimate benefit of patients in multiple contexts in ANZ and beyond.

## Conflicts of Interest

The author declares no conflicts of interest.

## Data Availability

Data sharing not applicable—no new data generated, or the article describes entirely theoretical research.
